# ﻿Anthemissect.Hiorthia (Asteraceae) on Kriti Island, Greece: high ploidy levels and a new species

**DOI:** 10.3897/phytokeys.229.102703

**Published:** 2023-07-13

**Authors:** Katerina Goula, Theophanis Constantinidis

**Affiliations:** 1 Section of Ecology & Systematics, Department of Biology, National and Kapodistrian University of Athens, Panepistimiopolis, 15784 Athens, Greece National and Kapodistrian University of Athens Athens Greece

**Keywords:** Anthemideae, chromosomes, Greek endemic, karyology, Mediterranean area, taxonomy

## Abstract

A morphological and karyological investigation of the Anthemissect.Hiorthia representatives of Kriti (Greece) revealed that three different species are found on the island, all endemic, and each characterised by a different ploidy level based on the haploid series of *x* = 9. *Anthemisabrotanifolia*, the species with the widest distribution, is tetraploid with 2*n* = 4*x* = 36. *A.samariensis*, a local endemic of the Lefka Ori, was found being decaploid, with 2*n* = 10*x* = 90, the highest number ever recorded in *Anthemis.* The recently discovered population on Mt. Kedros (south-central Kriti) is morphologically distinct from all the *Anthemis* entities growing on Kriti; it also differs from the variable and widespread *A.cretica* group. It is here described as a new species, *A.pasiphaes* Goula & Constantinidis. It is a hexaploid, with 2*n* = 6*x* = 54. All chromosome numbers are reported for the first time. Polyploidy might have acted as a reproductive barrier among these perennial species, complementing isolation by spatial distance and evolutionary divergence. Further, it might have contributed adaptation advantages to these three predominately mountain species.

## ﻿Introduction

Among the biodiversity hotspots in the Mediterranean Basin, Kriti or Crete (Greece) has a prominent position (Médail 2017). No fewer than 2240 plant taxa have been recorded on this large continental island (Kougioumoutzis et al. 2020), where the percentage of endemism is the highest known in Greece (Dimopoulos et al. 2013). The island’s geographical isolation, permanent since the early Pliocene (Greuter 1972; Sakellariou and Galanidou 2016), combined with mountain isolation due to Kriti’s uplifting and rugged topography, have played a significant role in plant endemism (Legakis and Kypriotakis 1994). The mountains of Kriti, in particular, have served both as refugia for old montane species and as cradles for plant diversification (Greuter 1972; Trigas et al. 2013).

One of the richest families in endemics on the island is Asteraceae, with at least 29 taxa endemic to Kriti, 13 of them restricted to mountainous areas (see Strid 2016b). With respect to the genus *Anthemis*, there are 14 taxa on the island (Strid 2016b; Goula and Constantinidis 2021); five of them, i.e., *A.abrotanifolia* (Willd.) Guss., *A.filicaulis* (Boiss. & Heldr.) Greuter, *A.glaberrima* (Rech.f.) Greuter, *A.samariensis* Turland, and *A.tomentella* Greuter, being regional endemics confined to Kriti and the nearby islets (Strid 2016b). Anthemissect.Hiorthia (DC.) R.Fernandes, formed mainly by perennial species of high altitude (Oberprieler 1998), was represented up to now by two endemic species in Kriti: *A.abrotanifolia* and *A.samariensis*. The latter is the *Anthemis* species that was most recently described on the island (Turland 2008). This chasmophyte was initially found in only two adjacent but distinct localities in the Lefka Ori, the richest area of Kriti in numbers of local endemic taxa (Montmollin and Iatrou 1995). The two subpopulations seem to face no threats by human activities or grazing. However, the species was assessed as “Vulnerable” according to the IUCN Red List criteria, due to its very restricted distribution area (Turland 2009). Five years after the description of *A.samariensis*, another population of a perennial *Anthemis* was discovered by Vangelis Papiomytoglou on Mt. Kedros, approximately 58 km as the crow flies ESE of the type locality of *A.samariensis* in the Lefka Ori. It was reported by Strid and Tan (2017) as conspecific with *A.samariensis*. Mt. Kedros is located in the central-southern part of Kriti, reaching 1776 m a.s.l., and is closer to Mt. Psiloritis (2456 m, the highest mountain of Kriti) than to the Lefka Ori.

In 2018, during field work focused on the taxonomic diversity of Greek *Anthemis*, the first author visited Mt. Kedros to collect material from this particular population. When this material was compared to *A.samariensis* from the *locus classicus*, the extent of the noticed morphological divergence led us to a more thorough examination of the samples. In addition, a karyological survey of the Mt. Kedros population, as well as those of *A.abrotanifolia* and *A.samariensis*, was carried out in order to explore and understand chromosome diversity and ploidy levels of all A.sect.Hiorthia members found in Kriti. The results are presented in this study.

## ﻿Materials and methods

Plant material, which included flowering and fruiting samples, was collected during two field trips on Mt. Kedros, in spring and summer of 2018. Dried specimens prepared from these samples were deposited in ATHU (the acronym follows Thiers 2022, continuously updated) and were examined thoroughly, in comparison with specimens of *Anthemissamariensis* from its *locus classicus* preserved at the herbarium of the Mediterranean Agronomic Institute of Chania (MAIC). The morphological diversity within the A.sect.Hiorthia members was also studied based on specimens collected between 2017 and 2021 (Goula, unpublished material), as well as specimens and digital specimen images provided by the herbaria ATH, ATHU, B, BM, BR, E, GOET, JE, K, MNHN, MO, NHMC, P, TAU, TAUF, UPA, W, WAG, and WU. The concept of the *A.cretica* entities and the protologues of subspecific taxa attributed to this name, together with descriptions and nomenclatural comments, were studied in historic and recent literature (Linnaeus 1737, 1753, 1763; Bieberstein 1808; Boissier 1875; Eig 1938; Fernandes 1975; Grierson and Yavin 1975; Fernandes 1976; Franzén 1986; Franzén 1991; Greuter et al. 2003; Greuter 2006+; Turland 2008; Strid 2016a, b).

In order to investigate the chromosome numbers of Anthemissect.Hiorthia of Kriti, mature achenes originating from populations of *A.abrotanifolia* on Mt. Psiloritis, *A.samariensis* on the Lefka Ori and plants of Mt. Kedros were cultivated in pots at the facilities of the Department of Biology, National and Kapodistrian University of Athens. Root tips from the seedlings were treated with a combined cycloheximide 0.0009% and 8-hydroxyquinoline 0.0006% solution for three hours, fixed in Carnoy’s solution for at least 24 hours and stored in ethanol 70% at -20 °C. To obtain photographs of metaphase plates, root tips were hydrolyzed in HCl 1N at 60 °C for 12 minutes, placed in Feulgen stain for up to two-and-a-half hours and squashed over microscope slides with a few drops of acetic acid 45%. Idiograms were constructed from photographs of at least three different metaphase plates (Goula and Constantinidis 2021). Construction of the idiograms was conducted using the KaryoType software, ver. 2.0 (Altınordu et al. 2016).

## ﻿Results

### ﻿Morphology

The morphological characters of the population on Mt. Kedros made it stand out as different from all known taxa of Anthemissect.Hiorthia from Kriti. Its closest relative, regarding morphology, seems to be *A.samariensis*, although it also appears to share common features with taxa of the variable *A.cretica* group from the Greek mainland and Anatolia. The morphological differences among members of A.sect.Hiorthia of Kriti are summarised in Table 1. Anthemiscreticasubsp.cretica, the member of the *A.cretica* group morphologically and geographically nearest to the Mt. Kedros population, has also been added for comparison reasons.

**Table 1.** Morphological differences between the Anthemissect.Hiorthia members of Kriti Island and A.creticasubsp.cretica from Peloponnisos (Greece). All measurements in mm.

		*Anthemisabrotanifolia*	*Anthemissamariensis*	Mt. Kedros population	Anthemiscreticasubsp.cretica
Stem indumentum		sericeous	usually glabrous to subglabrous	woolly	sericeous
Leaf indumentum		sericeous	villous	woolly	sericeous
Lobed leaves along stem		present	usually absent	present	present
Leaf dimensions	Outline	15–40 × 10–20	20–45 × 15–20	20–30 × 14–16	15–40 × 8–15
	Petiole width	0.3–0.6	1.5–2	0.5–1	0.3–0.6
	Ultimate lobes width	0.3–0.8	1–1.8	0.7–1.5	0.3–0.6
Number of primary leaf segments		5–7	usually 7	usually 7	(7–)9–15
Involucral bracts dimensions	Length	3–5	4–6	3.5–5	2–5
	Width	0.8–1.4	2–2.5	1.2–1.5	1.3–2
	Margin width	ca. 0.1	0.3–0.5	0.1–0.3	ca. 0.1
Receptacular scales	Length	3–3.5	6.5–7	4–6	3.5–4
	Width	0.9–1.3	ca. 1	0.7–1	ca. 1
	Apex	trucate to cuneate	emarginate	usually cuneate	cuneate
	Arista	0.1–0.2	ca. 1	1.5–2	0.5
Number of ligulate florets		0–8	8–14	14–20	14–16(–20)
Ligulate florets dimensions	Tube length	1.6–2	1.5–2	2–2.5	1.8–2
	Tube width	0.5–0.7	0.5–0.7	ca. 1	0.5–0.7
	Ligule width	1–2.5	5–6.5	3–5	3–4
Achene dimensions	Length	1.3–2	(2.1–)2.3–2.5(–2.8)	1.8–2.6	1.4–2
	Width	0.5–0.8	0.8–1	0.5–0.8	0.8–1
Pappus dimensions	Corona	0.1	0.2	0.2–0.4	0.3–0.6
	Auricle	absent	1.5	0.5–1	0.3–0.6
Pappus on achenes of ligulate florets		acute, dentate rim with 2–3 larger teeth	3-lobed auricle	denticulate auricle	denticulate to lobed oblique corona

In addition to the morphological differences of Table 1, the Mt. Kedros *Anthemis* and the variable *A.cretica* group also exhibit two noteworthy qualitative dissimilarities, as follows: a) the stem leaves of the Mt. Kedros population are clearly petiolate, whereas the stem leaves of *A.cretica* have a pair of lobes at or near their rachis base, thus appearing as almost sessile, and b) the receptacular scales of the Mt. Kedros population are aristate, in contrast to the acute to acuminate scales of the *A.cretica* group. A comparison of the Mt. Kedros population with the morphologically most relevant *A.cretica* subspecies, i.e., A.creticasubsp.cretica, and also A.creticasubsp.carpatica (Willd.) Grierson and A.creticasubsp.cassia (Boiss.) Grierson, reveals additional qualitative and quantitative differences. Anthemiscreticasubsp.cretica, particularly those populations from the mountain areas of Peloponnisos that are geographically closest to Kriti, differ further in that they bear a large number of primary leaf segments (up to 15 vs. usually 7 in the Mt. Kedros *Anthemis*) with much narrower ultimate lobes (0.3–0.6 mm vs. 0.7–1.5 mm in the Mt. Kedros *Anthemis*), narrower involucres (7–11 mm vs. 10–12 mm in the Mt. Kedros *Anthemis*), a conical, acute receptacle, smaller outer and inner achenes (1.4–2 mm with a pappus up to 0.6 mm vs. 1.8–2.6 mm with a pappus up to 1 mm in the Mt. Kedros *Anthemis*), and in the shape of the corona on the achenes of the ligulate florets (see Fig. 4, F1 & F3). Anthemiscreticasubsp.carpatica differs from the Kedros population in its indumentum, varying from totally glabrous to slightly sericeous, and the completely different pappus shape, consisting of an acute rim or a very small, ca. 0.2 mm corona, with no auricle. Anthemiscreticasubsp.cassia from E and S Anatolia, Syria and Lebanon (Grierson and Yavin 1975; Greuter 2006+) has wider ultimate leaf lobes (usually 2–3.5 mm vs. 0.7–1.5 mm in the Mt. Kedros *Anthemis*), shorter pappus on the achenes (0.5 mm vs. up to 1 mm in the Mt. Kedros *Anthemis*), whereas its involucral bracts margin may be pale, in contrast to the Mt. Kedros population with its always dark bract margin.

### ﻿Karyology

**Figure 1.**
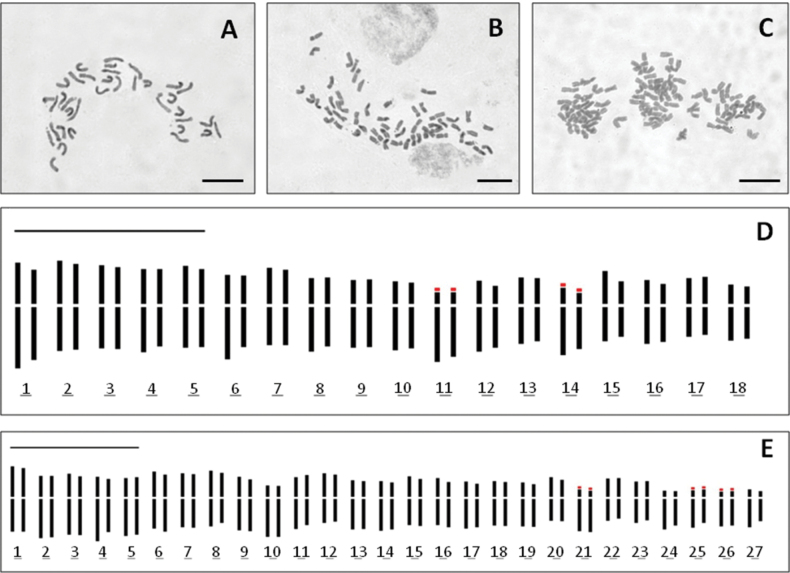
Metaphase plates and idiograms of Anthemissect.Hiorthia members in Kriti **A***A.abrotanifolia* from Mt. Psiloritis **B***Anthemis* population from Mt. Kedros **C***A.samariensis* from Lefka Ori **D** idiogram of *A.abrotanifolia***E** idiogram of Mt. Kedros population. Scale bars: 10 μm.

The examination of metaphase plates of the three Anthemissect.Hiorthia members of Kriti revealed three distinct ploidy levels. *Anthemisabrotanifolia* from Mt. Psiloritis was found to be tetraploid with 2*n* = 4*x* = 36 (Fig. 1a). The Mt. Kedros population was found to be hexaploid with 2*n* = 6*x* = 54 (Fig. 1b). All the cultivated plants of *A.samariensis* from the Lefka Ori were found to be decaploid with the surprising number of 2*n* = 10*x* = 90 (Fig. 1c). The chromosome numbers of all taxa are reported here for the first time. The decaploid chromosome level was unknown up to now in *Anthemis* and is therefore reported here as new. The large chromosome number of *A.samariensis* complicated its idiogram construction and the detailed study of chromosome morphology. The idiograms of *A.abrotanifolia* and the Mt. Kedros population are shown in Fig. 1 (1d and 1e, respectively). The karyotype of *A.abrotanifolia* consists of 22 metacentric, ten submetacentric and four subtelocentric chromosomes that bear satellites (karyotype formula: 2*n* = 4*x* = 22m + 10sm + 4st^4sat^). The karyotype of the Mt. Kedros population consists of 24 metacentric, 18 submetacentric and 12 subtelocentric chromosomes. Six of the latter bear satellites (karyotype formula: 2*n* = 6*x* = 24m + 18sm + 12st^6sat^). In Greek representatives of A.sect.Hiorthia, tetraploids are more common. The karyotypes of the Greek tetraploid (2*n* = 4*x* = 36) *A.cretica* populations (various subspecies) also consist of 24 metacentric chromosomes, but usually with four submetacentric and eight subtelocentric chromosomes that bear four to six satellites (Goula, unpublished data). The only hexaploid *A.cretica* subspecies in Greece (A.creticasubsp.carpatica) has a different karyotype structure from that of the Mt. Kedros population, formulated as 2*n* = 6*x* = 28m + 14sm + 10st^2sat^ + 2t^2sat^. Anthemiscreticasubsp.cretica from Peloponnisos is tetraploid with a karyotype formula of 2*n* = 4*x* = 24m + 6sm + 6st^6sat^ (Goula, unpublished data).

## ﻿Discussion

**Figure 2.**
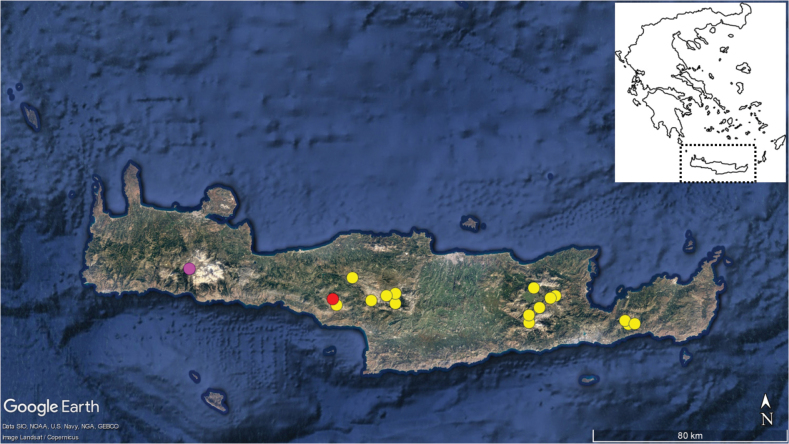
Distribution of Anthemissect.Hiorthia on Kriti Island. Pink dot: *A.samariensis*, red dot: *A.pasiphaes*, yellow dots: *A.abrotanifolia*. Based on Strid (2016b) and additional specimens in ATHU, MAIC and NHMC. Background map data: Google, SIO, NOAA, U.S. Navy, NGA, GEBCO.

Τhe morphological distinction of the Mt. Kedros *Anthemis* population from the other two members of A.sect.Hiorthia of Kriti and the related *A.cretica* group, coupled with the different ploidy levels revealed in our study, allow the recognition of a new species described here as *Anthemispasiphaes* Goula & Constantinidis (see below). According to current knowledge, this new species is endemic to Mt. Kedros (Fig. 2) and adds a new narrow endemic to the flora of Kriti. As a rule, local and regional Greek endemics are more common in the southern parts of the country, particularly Kriti and Peloponnisos, following an increase of the endemism rate observed in a north to south direction (e.g., Georghiou and Delipetrou 2010). The center of *Anthemis* diversity is in SW Asia (Lo Presti et al. 2010) and that of the heterogenous *A.cretica* group, to which both *A.samariensis* and *A.pasiphaes* presumably link, is apparently in Anatolia (Franzén 1991).

In order to decide on the taxonomic position of *Anthemispasiphaes* we considered the discussion provided by Turland (2008) in the case of *A.samariensis* and the arguments presented below. We concluded that *A.pasiphaes* would better be kept as a separate species and not fall under the variability of the *A.cretica* group for the reasons explained below.

1. Geological evidence indicates that Kriti has been permanently isolated from continental Greece and SW Asia since the early Pliocene, about 4 mya. By that time, it appears that the *Anthemiscretica* clade had already been separated from the rest of *Anthemis*, although diversification within its clade began about 2 mya (Lo Presti and Oberprieler 2009). It is plausible that the speciation of the *A.* sect. *Hiorthia* members on Kriti (*A.abrotanifolia*, *A.pasiphaes*, *A.samariensis*) was the result of geographical vicariance events of a local clade. Allopatric speciation due to isolation in mountain ranges drove diversification in other groups of Asteraceae rich in endemic species as well (e.g. *Centaurea*, López-Vinyallonga et al. 2015).
2. The *Anthemiscretica* assemblage is a diverse group of taxa with a complicated phylogenetic, taxonomic, and nomenclatural history. Concepts related to the group have changed over the decades. Most of the nowadays accepted subspecific entities classified under *A.cretica* appeared as new combinations in Grierson (1975). Grierson (1974) delved into the labyrinth of the historic literature on *A.cretica* and elucidated several species names, which, as he characteristically mentioned “had suffered a history of misapplication”. However, the origin and taxonomic category of the *A.cretica* lectotype specimen (Herb. Clifford: 415, *Anthemis* 2, BM000647187!) remains unresolved. Linnaeus (1763), cited “Habitat in Italia Helvetia” as the origin area of the species (under *A.montana* L., an illegitimate replacement name for *A.cretica*), whereas Franzén (1986), after examining material from the entire *A.cretica* distribution area, considered the East Aegean Islands as a more probable origin area. Furthermore, it is not yet clarified whether the Linnaean lectotype specimen was collected from a wild population or belongs to a cultivated specimen, i.e., it possesses possible distorted characters. According to the points presented above, the concept of the *A.cretica* needs further elucidation and is rather built on a shaky foundation. As of today, the number and rank of taxa within the *A.cretica* collective species are not yet fully resolved and large databases are not in full accordance. Euro+Med Plantbase (Greuter 2006+), for example, accepts 25 subspecies within *A.cretica*, compared to the World Flora Online (WFO 2022), which accepts 23 subspecies. Morphological differences between infraspecific *A.cretica* entities and certain corresponding species of *A.* sect. *Hiorthia* may be quite vague. For example, our field experience with some *Anthemis* populations of N Greece, particularly those of high mountains, makes distinguishing between *A.cretica* subsp. *carpatica* and *A.pindicola* Halácsy problematic. Grierson (1975) himself characterised his *A.cretica* group classification as “possibly oversimplified” and “tentative” and underlined the necessity of a biosystematic study within the group. The inclusion of new taxa within an even broader *A.cretica* complex would add intricacy to the whole structure. Cutting-edge molecular tools, when used, would presumably help in elucidating phylogeny and would offer a classification scheme in compliance with evolutionary patterns.
3. Polyploidy is one of the reproductive barriers responsible for isolating plant populations, and at the same time a driving force of speciation (Rieseberg and Willis 2007). Within the Mediterranean Basin in particular, polyploidy has played a major role in the diversification of several plant genera (e.g., Tomasello and Oberprieler 2022). In *Anthemis*, polyploidy has been recorded almost exclusively in *A.* sect. *Hiorthia*, where it is evolutionarily important (Kuzmanov et al. 1981). Hexaploids (2 *n* = 6 *x* = 54) and octoploids (2 *n* = 8 *x* = 72) have been recorded in only one representative of this section: *A.cretica* subsp. *carpatica* (Küpfer 1974; Baltisberger 1993). In Greece, tetraploid cytotypes (2 *n* = 4 *x* = 36), along with the typical diploid number (2 *n* = 2 *x* = 18), are more common, but hexaploid cytotypes also occur within the *A.cretica* complex (Goula and Constantinidis 2019). In our case, the three different, high ploidy levels of the *Anthemis* on Kriti (*A.abrotanifolia*, *A.pasiphaes*, *A.samariensis*) corroborate their morphological differentiation forming reproductive barriers and thus supporting their specific rank.

Incidence of polyploidy in plants depends on various factors, among them the climate and the life form (Rice et al. 2019). High chromosome numbers are more common within certain families, e.g., Asteraceae (Semple and Watanabe 2009). Within tribe Anthemideae, in particular, several genera have been reported to form extensive polyploid complexes as, e.g., *Leucanthemum* with ploidy levels varying from 2*x* to 22*x* (see Oberprieler et al. 2009). The higher genetic diversity provided through polyploidy improves environmental adaptation and tolerance, resulting in the ability of plants to colonise and be successful in harsh environmental contexts (Meudt et al. 2021). Polyploidy in the three Anthemissect.Hiorthia representatives of Kriti, restricted to calcareous stony slopes (*A.abrotanifolia*) or cliffs in mountain regions (*A.pasiphaes* and *A.samariensis*), seems to offer an advantage in adapting to and surviving in hostile habitats.

### ﻿Taxonomic treatment

#### ﻿ Anthemis pasiphaes

Taxon classificationPlantaeAsteralesAsteraceae

Goula & Constantinidis sp. nov.

3D38E591-CAFD-570A-AD1F-B33D03201A78

urn:lsid:ipni.org:names:77323168-1

##### Diagnosis.

Member of Anthemissect.Hiorthia related to *A.samariensis*, but differing in its woolly indumentum, presence of lobed leaves on flowering stems, longer aristae (1.5–2 mm) on receptacular scales, and presence of denticulate auricle on achenes of ligulate florets.

##### Type.

**Figure 3.**
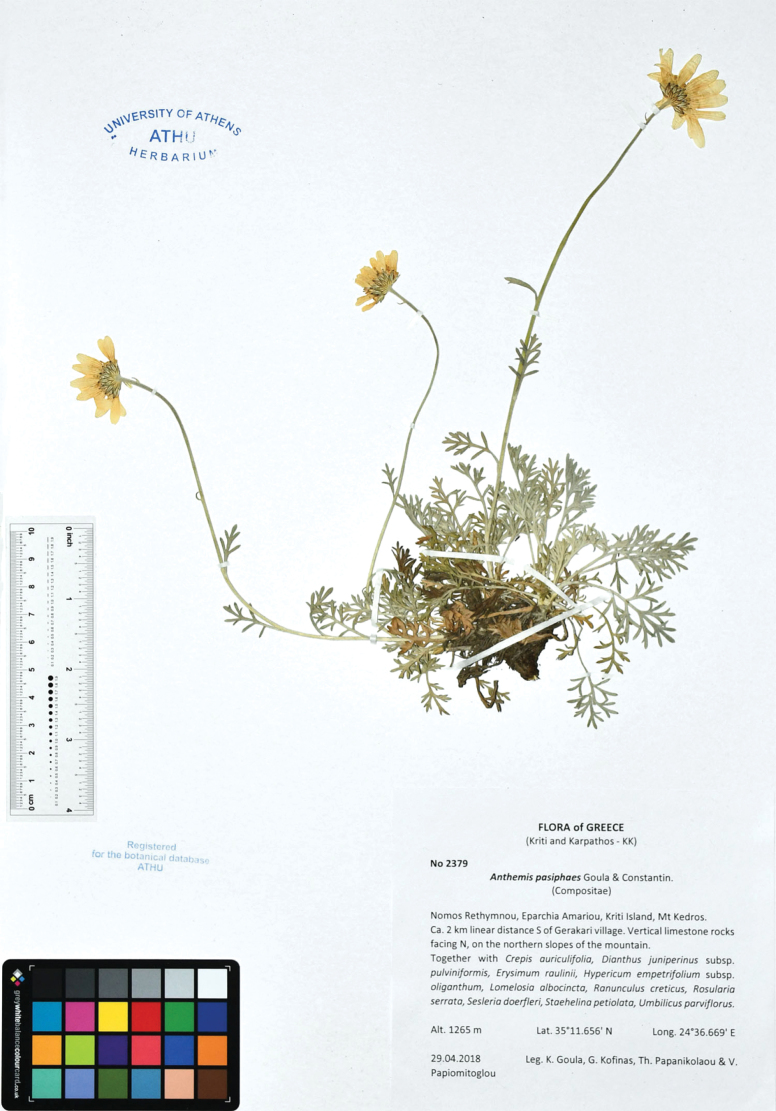
Holotype of *Anthemispasiphaes* Goula & Constantinidis (ATHU).

**Figure 4.**
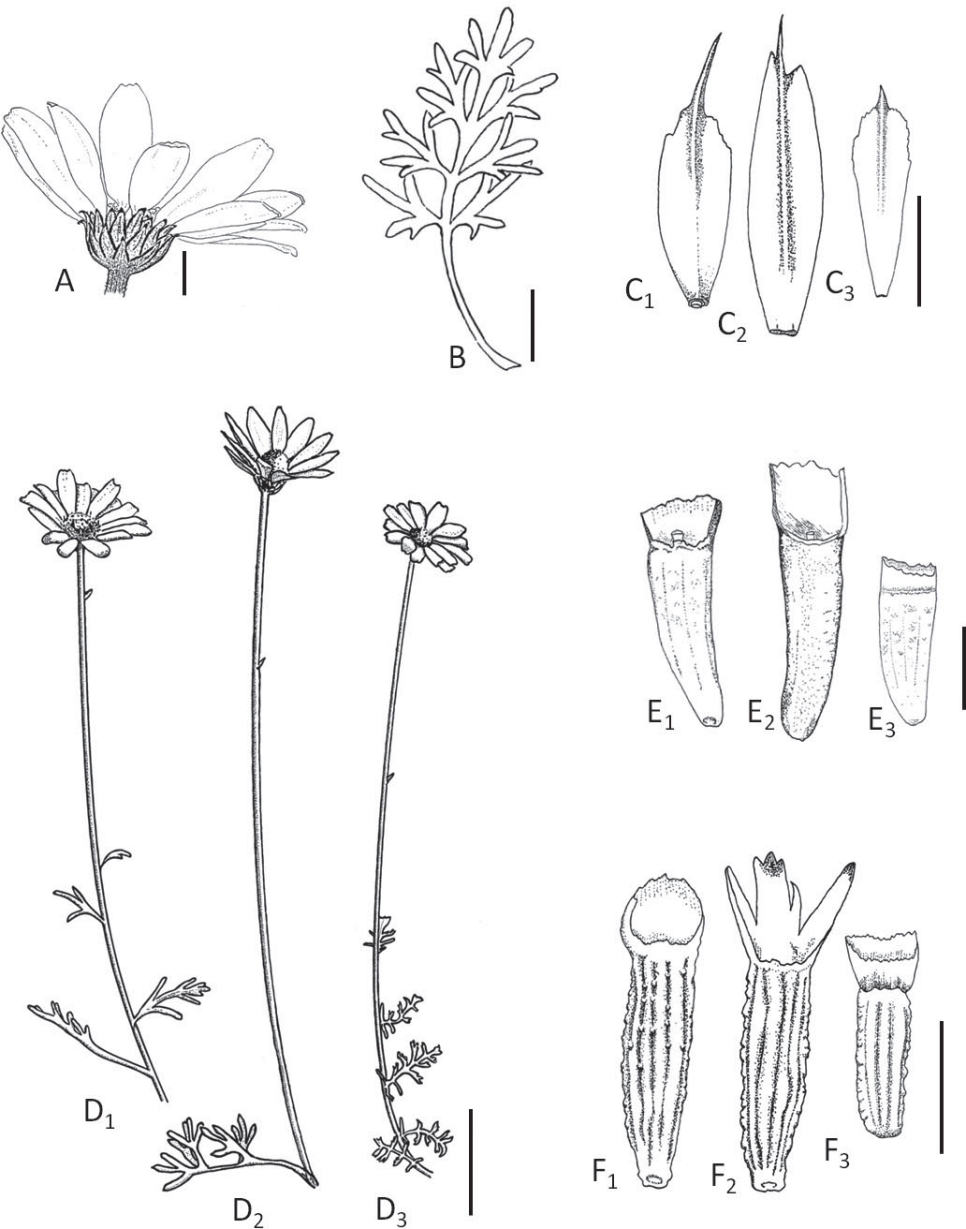
*Anthemispasiphaes* Goula & Constantinidis and comparison with *A.samariensis* and A.creticasubsp.cretica**A** capitulum **B** leaf **C** receptacular scales **D** flowering stems **E** achenes of disk florets **F** achenes of ligulate florets **C_1_**–**F_1_***A.pasiphaes***C_2_**–**F_2_***A.samariensis***C_3_**–**F_3_**A.creticasubsp.cretica. Scale bars: 5 mm (**A**); 1 cm (**B**); 2 mm (**C, F**); 3 cm (**D**); 1 mm (**E**). Drawn by N.A. Katsaros. *A.pasiphaes* was drawn from the holotype (Goula et al. 2379) and Goula & Katsaros (2644), both in ATHU, *A.samariensis* from material collected from the type locality (Ap. Kal. 9685, MAIC) and A.creticasubsp.cretica from material collected on Mt. Parnonas (Goula & Katsaros 2610, ATHU).

**Figure 5.**
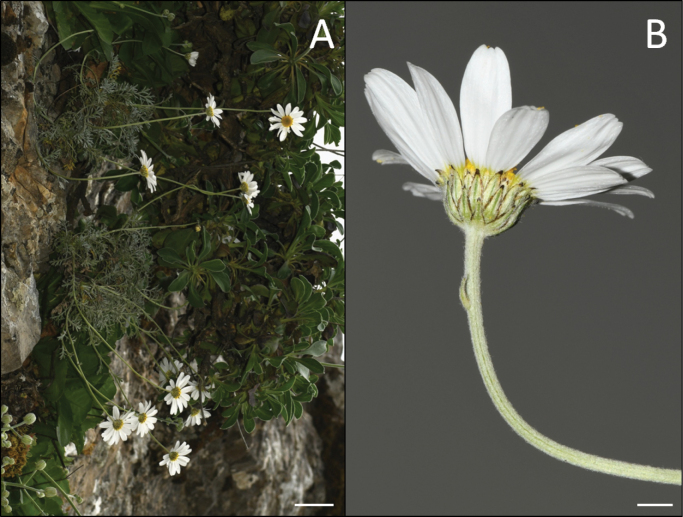
*Anthemispasiphaes* at its locus classicus **A** plant growing on a vertical rock **B** capitulum. Photo taken on 29.04.2018 by K. Goula. Scale bars: 5 cm (**A**); 5 mm (**B**).

Greece. Kriti: Nomos Rethimnou, Eparchia Amariou. Mt. Kedros, ca. 2 km linear distance S of Gerakari village, vertical limestone rocks facing N, on the northern slopes of the mountain, 1265 m a.s.l., 35°11'N, 24°36'E, 29 April 2018, Goula, Kofinas, Papanikolaou & Papiomytoglou 2379 (holotype, ATHU). Figs 3–5.

##### Description.

Perennial herb with stock covered in last year’s leaf sheaths. Indumentum woolly, ± appressed, hairs medifixed. Glands present in most parts of plant. Stems simple or branched; leafy non-flowering shoots present at anthesis. Flowering stems decumbent to erect, simple, 10–25 cm tall, angled, woolly, greyish-green, with successively smaller and less dissected leaves up to middle, and entire, scale-like leaves up to almost below capitulum. Leaves somewhat aromatic with golden stalked glands on leaf surface, greyish-green, up to 6 cm long, with both surfaces woolly; petiole up to 3 cm long and 0.5–1 mm wide; leaf blade 2-pinnatisect, ovate in outline, 2–3 cm × ca. 1.5 cm; primary segments usually 7, each one divided into 2–5 ultimate lobes; ultimate lobes narrowly oblanceolate to obovate, 0.7–1.5 mm wide, apex subacute with minute cartilaginous cusp, usually hidden below the dense trichomes. Capitulum solitary, radiate. Involucre hemispherical, 10–12 mm wide. Involucral bracts imbricate, greyish-green, lanceolate, 3.5–5 × 1.2–1.5 mm, outer surface villous with dark green or dark brown midvein; margin dark brown, 0.1–0.3 mm wide, membranous, densely and minutely lacerate, apex dark brown to black, acute to acuminate. Receptacle hemispherical becoming hemispherical-conical, apex obtuse. Receptacular scales narrowly oblanceolate, navicular, 4–6 × 0.7–1 mm, scarious, apex usually cuneate or emarginate, midvein straw coloured, prominent, leading to arista (1–)1.5–2 mm long. Ligulate florets 14–20; tube green, cylindrical, 2–2.5 mm × ca. 1 mm; ligules patent at anthesis, later reflexed, white, oblong to oblong-obovate, 10–15 × 3–5 mm, spotted with sessile glands. Disk florets yellow, spotted with sessile glands; tube cylindric, 3–3.5 mm long (including the lobes), 0.5–0.8 mm wide; lobes 5, triangular, 0.5–0.7 mm long; lower part of disk florets swollen and spongy at maturity. Achenes straw-coloured, narrowly obconic-oblong. Achenes of disk florets weakly 4-angled, slightly curved, 1.8–2.5 mm long, excluding pappus, 0.5–0.8 mm wide, more or less longitudinally ribbed; pappus oblique, forming short lacerate corona 0.2–0.4 mm wide and lacerate auricle adaxially; auricle scarious, 0.5–0.8 mm long, densely and finely longitudinally veined. Achenes of ligulate florets more curved and more prominently ribbed, 2.3–2.6 mm long excluding pappus, surface characters as in achenes of disk florets, but additionally sessile glands present; pappus as in achenes of disk florets, but auricle entire, 0.8–1 mm long, with lacerate apex. 2*n* = 6*x* = 54.

##### Distribution and habitat.

*Anthemispasiphaes* is apparently endemic to Mt. Kedros, restricted to its northern part (Fig. 2). It grows on steep, calcareous cliffs, mostly inaccessible even to the numerous goats that graze the area. Currently known only from the type locality, at 1265 m a.s.l., but presumably also occurring higher up, on the same slope. It grows together with other endemics of Kriti, like *Crepisauriculifolia* Spreng., Dianthusjuniperinussubsp.pulviniformis (Greuter) Turland, *Erysimumraulinii* Boiss., *Lomelosiaalbocincta* (Greuter) Greuter & Burdet, *Sesleriadoerfleri* Hayek, and *Staehelinapetiolata* (L.) Hilliard & B.L.Burtt.

##### Etymology.

The specific epithet derives from the female name Pasiphaë and consists of the Greek words *πάσι* (= all) and *φάος/φῶς* (= light), i.e., “she who illuminates everyone”. Pasiphaë was daughter of Helios (god of the Sun), wife of King Minos, Queen of Kriti and immortal, according to Greek mythology.

##### Phenology.

Flowering from late April to early June; fruiting from June to July.

##### Conservation status.

*Anthemispasiphaes* is currently known from the type locality only. This single population is considerably small, with no more than 50 individuals counted, and restricted to practically inaccessible cliffs. The species has not been recorded elsewhere, although there are several similar habitats around in Kriti, which is botanically one of the best explored regions of Greece (Strid and Tan 2017). Potentially suitable localities on Mt. Kedros have not revealed any additional populations so far. The presence of grazing animals in the area is very apparent, limiting the small population to very steep cliffs. Neither mature individuals nor leaf rosettes were observed in localities accessible to goats. A rock-climbing area on the southern slopes of Mt. Kedros is not a threat to *A.pasiphaes* at present; however, the northern part of the mountain is also suitable for rock-climbing activities that would potentially destroy the only known population. Αlthough we counted a very small number of mature plants, it is possible that more plants are present, very locally, on cliffs surrounding the *locus classicus*, given that they are not accessible to goats and retain enough moisture and some shade to permit uninterrupted growth of *A.pasiphaes.* If distribution and frequency of mature plants follow the same patterns we observed during field work, we may then estimate with some certainty that the total population of the species is fewer than 250 mature individuals. Therefore, the species meets the Criterion D (number of mature individuals <250) following the IUCN Guidelines for the assessment of taxa known only from the type locality (IUCN Standards and Petitions Committee 2022). The IUCN Red List category of Endangered seems suitable (EN D).

### ﻿A revised key to genus *Anthemis* and the related genus *Cota* from Kriti (including surrounding islets)

**Table:** 

1	Achenes compressed, rhombic in transverse section; leaf segments pectinate	**2**
–	Achenes not compressed, orbicular or sub-quadrangular in transverse section; leaf segments usually not pectinate	**3**
2	Receptacular scales straw colour at maturity; plant usually erect	***C.altissima***
–	Receptacular scales purplish-brown to almost black at maturity; plant usually procumbent	***C.melanolepis***
3	Plant annual, non-flowering shoots absent at anthesis	**4**
–	Plant perennial, non-flowering shoots present at anthesis	**13**
4	Receptacle without scales	**5**
–	Receptacle with scales present at least on upper part	**6**
5	Leaves somewhat fleshy, lobes obtuse; ligules absent; achenes caducous, with a ca. 0.5 mm long corona	**A.ammanthussubsp.ammanthus**
–	Leaves not fleshy, lobes acute; ligules occasionally present; achenes with a ca. 1 mm long corona, outer achenes persistent, inner caducous	***A.filicaulis***
6	Receptacle without scales in lower part; achenes cylindrical to turbinate, tuberculate, pappus absent	***A.cotula***
–	Receptacle with scales all over, at least when young; achenes with a different combination of characters	**7**
7	Receptacular scales hairy	**A.ammanthussubsp.paleacea**
–	Receptacular scales glabrous	**8**
8	Peduncles not or slightly clavate in fruit; at least inner achenes not firmly attached to receptacle	**9**
–	Peduncles clavate in fruit; achenes firmly attached to receptacle or involucre indurate at maturity	**11**
9	Plants slender; ligules not more than 5 mm or absent; margin of involucral bracts pale; achenes not or obscurely ribbed	**10**
–	Ligules always present, longer than 7 mm; involucral bracts usually with dark margin; achenes with 7–10 distinct ribs	***A.chia***
10	Ligules always present, pinkish at least beneath; receptacular scales linear-lanceolate; achenes with a fimbriate corona	***A.glaberrima***
–	Ligules absent or, if present, white; receptacular scales linear-setaceous; achenes with an entire to lacerate corona	***A.tomentella***
11	Stems erect; receptacle conical, elongated; achenes with a thickened marginal rim, pappus absent	**A.arvensissubsp.incrassata**
–	Stems prostrate to ascending; receptacle shortly conical; achenes with acute rim and a corona at least 0.3 mm long	**12**
12	Ligules absent; capitula discoid	**A.rigidasubsp.rigida**
–	Ligule present, white; capitula radiate	**A.rigidasubsp.ammanthiformis**
13	Involucre 4–7 mm long, ligules usually absent; disk florets yellow or pink; achenes without pappus or with a very short acute rim	***A.abrotanifolia***
–	Involucre 8–12 mm long, ligules present, large; disk florets yellow; achenes with a 0.5–1.5 mm long auricle	**14**
14	Flowering stems glabrous at least at middle part, leafless except for small, scale-like leaves; receptacular scales with an arista ca. 1 mm long; achenes of ligulate florets with a 3-lobed auricle	***A.samariensis***
–	Flowering stems woolly, bearing dissected leaves usually up to middle; receptacular scales with an arista 1.5–2 mm; achenes of ligulate florets with an entire auricle	***A.pasiphaes***

## Supplementary Material

XML Treatment for Anthemis pasiphaes

## ﻿Additional information

### Author ORCIDs

Katerina Goula https://orcid.org/0000-0001-9207-3570

Theophanis Constantinidis https://orcid.org/0000-0001-9704-3864

### Data availability

All of the data that support the findings of this study are available in the main text or Supplementary Information.

## References

[B1] Altınordu F Peruzzi L Yu Y He X (2016) A tool for the analysis of chromosomes: KaryoType. Taxon 65(3): 586–592. 10.12705/653.9

[B2] Baltisberger M (1993) Zytologische Untersuchungen an Compositen aus Albanien. Candollea 48(2): 437–448.

[B3] Bieberstein FAM (1808) Flora TauricoCaucasica, Vol. 2. Charkouiae, Typis Academicis, 1–478.

[B4] Boissier E (1875) Flora Orientalis sive enumeratio plantarum in oriente a Graecia et Aegypto ad Indiae fines hucusque observatarum 3. H. George, Genevae et Basileae, Lugduni, 1–1033.

[B5] Dimopoulos P Raus Th Bergmeier E Constantinidis Th Iatrou G Kokkini S Strid A Tzanoudakis D (2013) Vascular plants of Greece: An annotated checklist. Botanischer Garten und Botanisches Museum Berlin-Dahlem, Berlin; Hellenic Botanical Society, Athens, 1–372. https://www.jstor.org/stable/i24365374

[B6] Eig A (1938) Taxonomic studies on the oriental species of the genus *Anthemis*. Le Journal de Botanique 1(2): 161–225.

[B7] Fernandes R (1975) Identification, typification, affinités et distribution géographique de quelques taxa Européens du genre *Anthemis* L. Anales del Instituto Botánico A. J. Cavanilles 32(2): 1409–1488.

[B8] Fernandes R (1976) *Anthemis* L. In: Tutin TG Heywood VH Burges NA Moore DM Valentine DH Walters SM Webb DA (Eds) Flora Europaea Volume 4.Cambridge University Press, Cambridge, 145–159.

[B9] Franzén R (1986) *Anthemiscretica* (Asteraceae) and related species in Greece. Willdenowia 16(1): 35–45. https://www.jstor.org/stable/3996297

[B10] Franzén R (1991) *Anthemis*. In: Strid A Tan K (Eds) Mountain Flora of Greece 2.Edinburgh University Press, Edinburgh, 420–431.

[B11] Georghiou K Delipetrou P (2010) Patterns and traits of the endemic plants of Greece. Botanical Journal of the Linnean Society 162(2): 130–422. 10.1111/j.1095-8339.2010.01025.x

[B12] Goula K Constantinidis Th (2019) Karyological studies in Greek *Anthemis* s.l. (Anthemideae, Asteraceae). In: Bareka P, Domina G, Kamari G (Eds) Proceedings of the 16th OPTIMA Meeting, Organization for the Phyto-taxonomic investigation of the Mediterranean area, Palermo, Italy, 134 pp.

[B13] Goula K Constantinidis Th (2021) Taxonomic diversity and karyology of *Anthemisrigida* (Anthemideae, Asteraceae) in the Aegean, Greece. Phytotaxa 484(1): 129–143. 10.11646/phytotaxa.484.1.7

[B14] Greuter W (1972) The relict element of the flora of Crete and its evolutionary significance. In: Valentine DH (Ed.) Taxonomy, phytogeography and evolution.Academic Press, London, 161–177.

[B15] Greuter W (2006+) Compositae (pro parte majore). In: Greuter W, Raab-Straube E von (Eds) Compositae. Euro+Med Plantbase – the information resource for Euro-Mediterranean plant diversity. 10.3372/wi.36.36206

[B16] Greuter W Oberprieler C Vogt R (2003) The Euro+Med treatment of Anthemideae (Compositae) – generic concepts and required new names. Willdenowia 33(1): 37–43. 10.3372/wi.33.33102

[B17] Grierson AJC (1974) *Anthemis*. In: Davis PH (Ed.) Materials for a Flora of Turkey XXX: Compositae, I.Notes from the Royal Botanic Garden Edinburgh 33(2), 207–264.

[B18] Grierson AJC (1975) *Anthemis*. In: Davis PH (Ed.) Materials for a Flora of Turkey XXXI: Compositae, II.Notes from the Royal Botanic Garden Edinburgh 33(3), 409–435.

[B19] Grierson AJC Yavin Z (1975) *Anthemis* L. In: Davis PH (Ed.) Flora of Turkey and the East Aegean islands.Edinburgh University Press, Edinburgh, 174–221.

[B20] IUCN Standards and Petitions Committee (2022) Guidelines for Using the IUCN Red List Categories and Criteria. Version 15.1. Prepared by the Standards and Petitions Committee. https://www.iucnredlist.org/documents/RedListGuidelines.pdf [Accessed 20/11/2022]

[B21] Kougioumoutzis K Kokkoris IP Panitsa M Trigas P Strid A Dimopoulos P (2020) Spatial phylogenetics, biogeographical patterns and conservation implications of the endemic flora of Crete (Aegean, Greece) under climate change scenarios. Biology (Basel) 9(8): 199. 10.3390/biology9080199 32751787 PMC7463760

[B22] Küpfer P (1974) Recherches sur les liens de parenté entre la flore orophile des Alpes et celle des Pyrénées. Boissiera 23: 1–322.

[B23] Kuzmanov B Thin NN Georgieva S (1981) A cytotaxonomic study on Bulgarian *Anthemis* species. Candollea 36: 19–76.

[B24] Legakis A Kypriotakis Z (1994) A biogeographical analysis of the island of Crete, Greece. Journal of Biogeography 21: 441–445. 10.2307/2845761

[B25] Linnaeus C (1737) Hortus Cliffortianus. Amstelaedami, 1–520.

[B26] Linnaeus C (1753) Species Plantarum 2. Laurentius Salvius, Holmiae, 561–1200.

[B27] Linnaeus C (1763) Species Plantarum, ed. 2, 2. Laurentius Salvius, Holmiae, 785–1684.

[B28] Lo Presti RM Oberprieler C (2009) Evolutionary history, biogeography and eco-climatological differentiation of the genus *Anthemis* L. (Compositae, Anthemideae) in the circum-Mediterranean area. Journal of Biogeography 36(7): 1313–1332. 10.1111/j.1365-2699.2009.02121.x

[B29] Lo Presti RM Oppolzer S Oberprieler C (2010) A molecular phylogeny and a revised classification of the Mediterranean genus *Anthemis* s.l. (Compositae, Anthemideae) based on three molecular markers and micromorphological characters. Taxon 59(5): 1441–1456. 10.1002/tax.595010

[B30] López-Vinyallonga S López-Pujol J Constantinidis Th Susanna A Garcia-Jacas N (2015) Mountains and refuges: Genetic structure and evolutionary history in closely related endemic *Centaurea* in continental Greece. Molecular Phylogenetics and Evolution 92: 243–254. 10.1016/j.ympev.2015.06.018 26151220

[B31] Médail F (2017) The specific vulnerability of plant biodiversity and vegetation on Mediterranean islands in the face of global change. Regional Environmental Change 17(6): 1775–1790. 10.1007/s10113-017-1123-7

[B32] Meudt HM Albach DC Tanentzap AJ Igea J Newmarch SC Brandt AJ Lee WG Tate JA (2021) Polyploidy on islands: Its emergence and importance for diversification. Frontiers in Plant Science 12: 637214. 10.3389/fpls.2021.637214 PMC7982887 33763097

[B33] Montmollin B Iatrou G (1995) Connaissance et conservation de la flore de l`île de Crète. Ecologia Mediterranea 21(1–2): 173–184. 10.3406/ecmed.1995.1765

[B34] Oberprieler C (1998) The Systematics of *Anthemis* L. (Compositae, Anthemideae) in W and C North Africa. Bocconea 9: 1–328.

[B35] Oberprieler C Himmelreich S Källersjö M Vallès J Watson LE Vogt R (2009) Anthemideae. In: Funk VA Susanna A Stuessy TF Bayer RJ (Eds) Systematics, evolution, and biogeography of Compositae.International Association for Plant Taxonomy, Vienna, Austria, 631–666.

[B36] Rice A Šmarda P Novosolov M Drori M Glick L Sabath N Meiri S Belmaker J Mayrose I (2019) The global biogeography of polyploid plants. Nature Ecology & Evolution 3(2): 265–273. 10.1038/s41559-018-0787-9 30697006

[B37] Rieseberg LH Willis JH (2007) Plant speciation. Science 317(5840): 910–914. 10.1126/science.1137729 17702935 PMC2442920

[B38] Sakellariou D Galanidou N (2016) Pleistocene submerged landscapes and Palaeolithic archaeology in the tectonically active Aegean region. In: Harff J Bailey G Lüth F (Eds) Geology and Archaeology: Submerged landscapes of the continental shelf.Geological Society of London Special Publication 411: 145–178. 10.1144/SP411.9

[B39] Semple JC Watanabe K (2009) A review of chromosome numbers in Asteraceae with hypotheses on chromosomal base number evolution. In: Funk VA Susanna A Stuessy TF Bayer RJ (Eds) Systematics, evolution, and biogeography of Compositae.International Association for Plant Taxonomy, Vienna, 61–72.

[B40] Strid A (2016a) Atlas of the Aegean flora, Part 1: Texts and Plates. Englera 33: 1–700. [Berlin: Botanic Garden and Botanical Museum Berlin, Freie Universität Berlin] https://www.jstor.org/stable/i40215597

[B41] Strid A (2016b) Atlas of the Aegean flora, Part 2: Maps. Englera 33: 1–878. [Berlin: Botanic Garden and Botanical Museum Berlin, Freie Universität Berlin] https://www.jstor.org/stable/i40216011

[B42] Strid A Tan K (2017) Recent progress in plant taxonomy and floristic studies in Greece. Botanica Serbica 41(2): 123–152.

[B43] Thiers B (2022 [continuously updated]) Index Herbariorum: A global directory of public herbaria and associated staff. https://sweetgum.nybg.org/science/ih/ [Accessed 4.10.2022]

[B44] Tomasello S Oberprieler C (2022) Reticulate evolution in the western Mediterranean mountain ranges: The case of the *Leucanthemopsis* polyploid complex. Frontiers in Plant Science 13: 842842. 10.3389/fpls.2022.842842 PMC9247603 35783934

[B45] Trigas P Panitsa M Tsiftsis S (2013) Elevational gradient of vascular plant species richness and endemism in Crete – The effect of post-isolation mountain uplift on a continental island system. PLoS ONE 8(3): e59425. 10.1371/journal.pone.0059425 PMC3595250 23555031

[B46] Turland NJ (2008) *Anthemissamariensis* (Asteraceae, Anthemideae), a new species from the mountains of W Kriti (Greece). Willdenowia 38(1): 61–69. 10.3372/wi.38.38103

[B47] Turland N (2009) *Anthemissamariensis*. In: Phitos D Constantinidis Th Kamari G (Eds) The Red Data Book of rare and threatened plants of Greece, Vol.1, A-D. Hellenic Botanical Society, Patras, 1–406.

[B48] WFO (2022) *Anthemiscretica* L. http://www.worldfloraonline.org/taxon/wfo-000002­3915 [Accessed on 20.11.2022]

